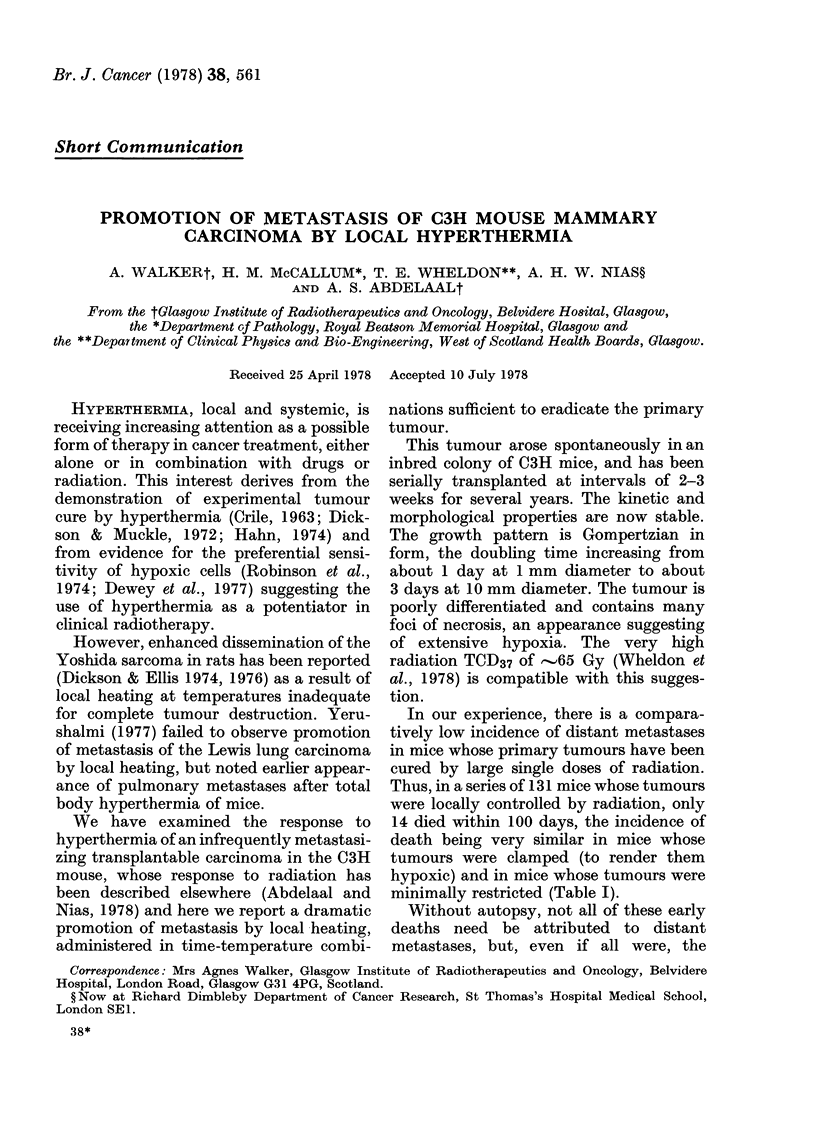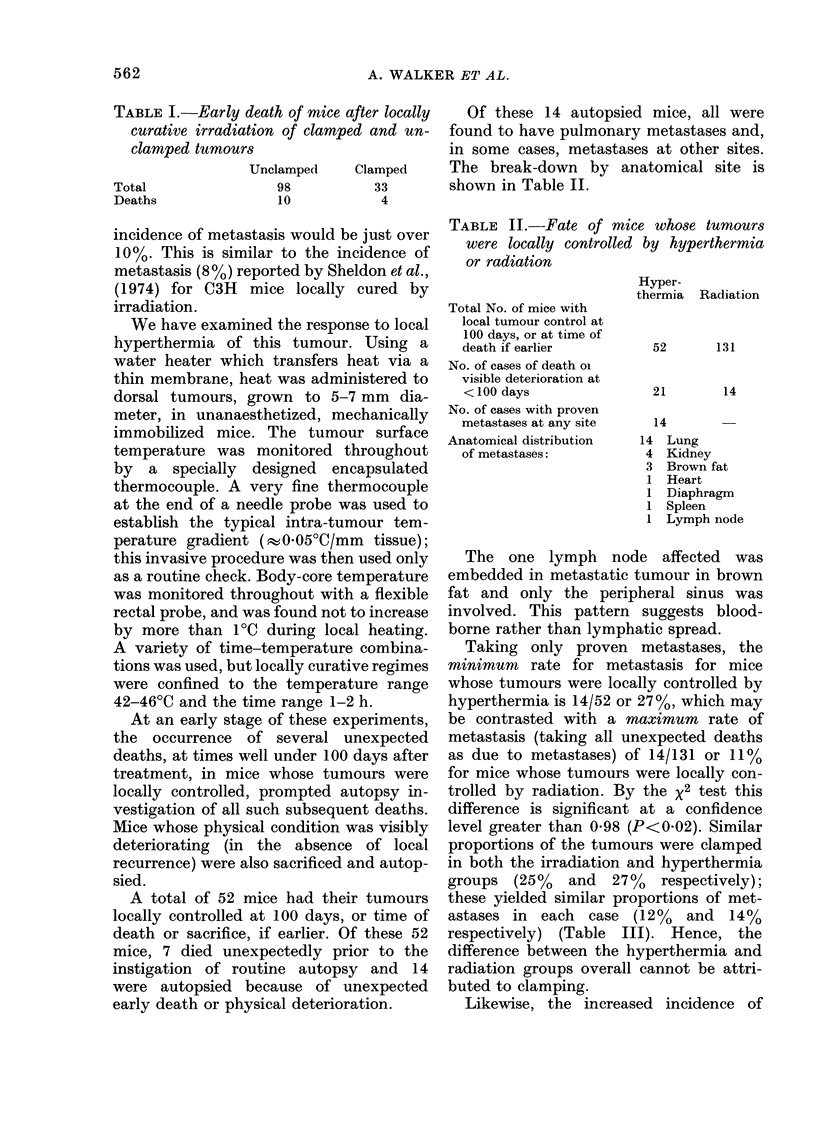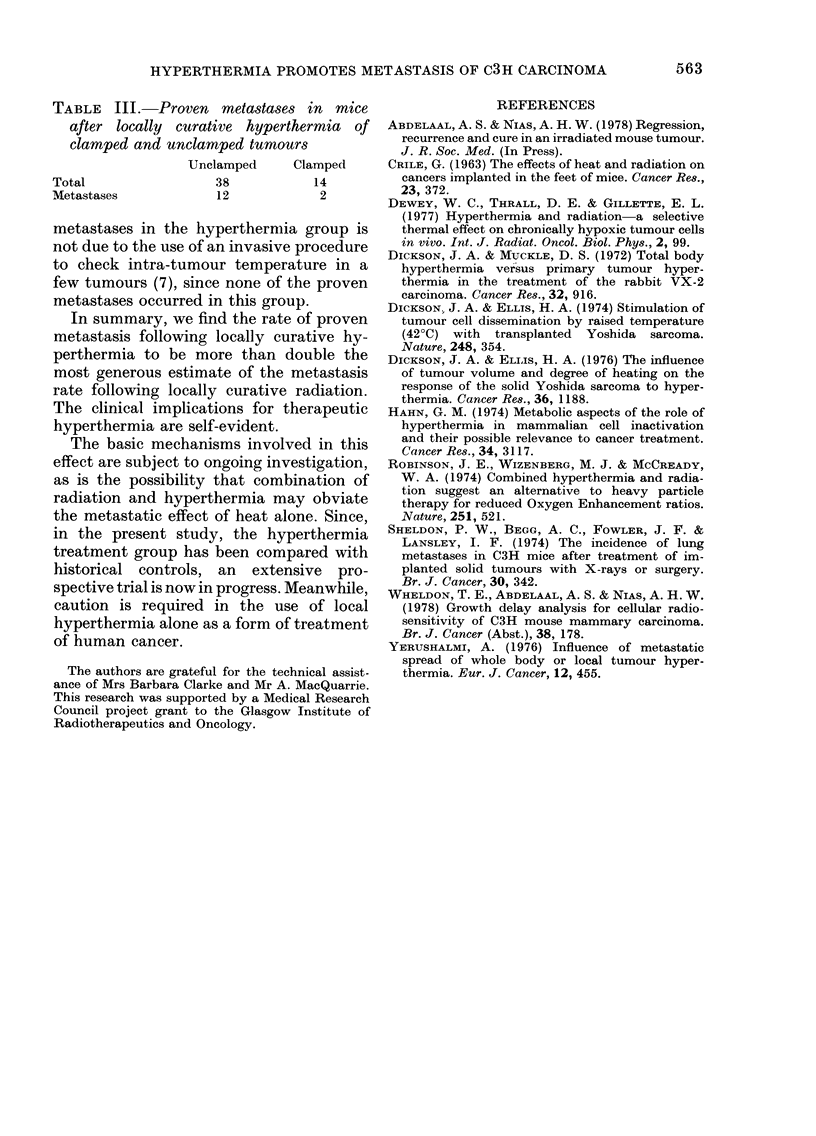# Promotion of metastasis of C3H mouse mammary carcinoma by local hyperthermia.

**DOI:** 10.1038/bjc.1978.246

**Published:** 1978-10

**Authors:** A. Walker, H. M. McCallum, T. E. Wheldon, A. H. Nias, A. S. Abdelaal


*Br. J. Cancer* (1978) **38**, 561

***Short Communication***

### PROMOTION OF METASTASIS OF C3H MOUSE MAMMARY CARCINOMA BY LOCAL HYPERTHERMIA

A. WALKER†, H. M. McCALLUM*, T. E. WHELDON**, A. H. W. NIAS§ AND A. S. ABDELAAL†

*From the †Glasgow Institute of Radiotherapeutics and Oncology, Belvidere Hosital, Glasgow, the *Department of Pathology, Royal Beatson Memorial Hospital, Glasgow and the **Department of Clinical Physics and Bio-Engineering, West of Scotland Health Boards, Glasgow.*

Received 25 April 1978 Accepted 10 July 1978

HYPERTHERMIA, local and systemic, is receiving increasing attention as a possible form of therapy in cancer treatment, either alone or in combination with drugs or radiation. This interest derives from the demonstration of experimental tumour cure by hyperthermia (Crile, 1963; Dickson & Muckle, 1972; Hahn, 1974) and from evidence for the preferential sensitivity of hypoxic cells (Robinson *et al.*, 1974; Dewey *et al.*, 1977) suggesting the use of hyperthermia as a potentiator in clinical radiotherapy.

However, enhanced dissemination of the Yoshida sarcoma in rats has been reported (Dickson & Ellis 1974, 1976) as a result of local heating at temperatures inadequate for complete tumour destruction. Yerushalmi (1977) failed to observe promotion of metastasis of the Lewis lung carcinoma by local heating, but noted earlier appearance of pulmonary metastases after total body hyperthermia of mice.

We have examined the response to hyperthermia of an infrequently metastasizing transplantable carcinoma in the C3H mouse, whose response to radiation has been described elsewhere (Abdelaal and Nias, 1978) and here we report a dramatic promotion of metastasis by local heating, administered in time-temperature combinations sufficient to eradicate the primary tumour.

This tumour arose spontaneously in an inbred colony of C3H mice, and has been serially transplanted at intervals of 2–3 weeks for several years. The kinetic and morphological properties are now stable. The growth pattern is Gompertzian in form, the doubling time increasing from about 1 day at 1 mm diameter to about 3 days at 10 mm diameter. The tumour is poorly differentiated and contains many foci of necrosis, an appearance suggesting of extensive hypoxia. The very high radiation $TCD_{37}$ of $\sim$65 Gy (Wheldon *et al.*, 1978) is compatible with this suggestion.

In our experience, there is a comparatively low incidence of distant metastases in mice whose primary tumours have been cured by large single doses of radiation. Thus, in a series of 131 mice whose tumours were locally controlled by radiation, only 14 died within 100 days, the incidence of death being very similar in mice whose tumours were clamped (to render them hypoxic) and in mice whose tumours were minimally restricted (Table I).

Without autopsy, not all of these early deaths need be attributed to distant metastases, but, even if all were, the

*Correspondence:* Mrs Agnes Walker, Glasgow Institute of Radiotherapeutics and Oncology, Belvidere Hospital, London Road, Glasgow G31 4PG, Scotland.

§Now at Richard Dimbleby Department of Cancer Research, St Thomas's Hospital Medical School, London SE1.

TABLE I.—*Early death of mice after locally curative irradiation of clamped and unclamped tumours*

| | Unclamped | Clamped |
|---|---|---|
| Total | 98 | 33 |
| Deaths | 10 | 4 |

incidence of metastasis would be just over 10%. This is similar to the incidence of metastasis (8%) reported by Sheldon *et al.*, (1974) for C3H mice locally cured by irradiation.

We have examined the response to local hyperthermia of this tumour. Using a water heater which transfers heat via a thin membrane, heat was administered to dorsal tumours, grown to 5–7 mm diameter, in unanaesthetized, mechanically immobilized mice. The tumour surface temperature was monitored throughout by a specially designed encapsulated thermocouple. A very fine thermocouple at the end of a needle probe was used to establish the typical intra-tumour temperature gradient ($\approx$0·05°C/mm tissue); this invasive procedure was then used only as a routine check. Body-core temperature was monitored throughout with a flexible rectal probe, and was found not to increase by more than 1°C during local heating. A variety of time–temperature combinations was used, but locally curative regimes were confined to the temperature range 42–46°C and the time range 1–2 h.

At an early stage of these experiments, the occurrence of several unexpected deaths, at times well under 100 days after treatment, in mice whose tumours were locally controlled, prompted autopsy investigation of all such subsequent deaths. Mice whose physical condition was visibly deteriorating (in the absence of local recurrence) were also sacrificed and autopsied.

A total of 52 mice had their tumours locally controlled at 100 days, or time of death or sacrifice, if earlier. Of these 52 mice, 7 died unexpectedly prior to the instigation of routine autopsy and 14 were autopsied because of unexpected early death or physical deterioration.

Of these 14 autopsied mice, all were found to have pulmonary metastases and, in some cases, metastases at other sites. The break-down by anatomical site is shown in Table II.

TABLE II.—*Fate of mice whose tumours were locally controlled by hyperthermia or radiation*

| | Hyper-thermia | Radiation |
|---|---|---|
| Total No. of mice with local tumour control at 100 days, or at time of death if earlier | 52 | 131 |
| No. of cases of death oı visible deterioration at <100 days | 21 | 14 |
| No. of cases with proven metastases at any site | 14 | — |
| Anatomical distribution of metastases: | 14 Lung | |
| | 4 Kidney | |
| | 3 Brown fat | |
| | 1 Heart | |
| | 1 Diaphragm | |
| | 1 Spleen | |
| | 1 Lymph node | |

The one lymph node affected was embedded in metastatic tumour in brown fat and only the peripheral sinus was involved. This pattern suggests blood-borne rather than lymphatic spread.

Taking only proven metastases, the *minimum* rate for metastasis for mice whose tumours were locally controlled by hyperthermia is 14/52 or 27%, which may be contrasted with a *maximum* rate of metastasis (taking all unexpected deaths as due to metastases) of 14/131 or 11% for mice whose tumours were locally controlled by radiation. By the $\chi^2$ test this difference is significant at a confidence level greater than 0·98 ($P<0{\cdot}02$). Similar proportions of the tumours were clamped in both the irradiation and hyperthermia groups (25% and 27% respectively); these yielded similar proportions of metastases in each case (12% and 14% respectively) (Table III). Hence, the difference between the hyperthermia and radiation groups overall cannot be attributed to clamping.

Likewise, the increased incidence of

TABLE III.—*Proven metastases in mice after locally curative hyperthermia of clamped and unclamped tumours*

| | Unclamped | Clamped |
|---|---|---|
| Total | 38 | 14 |
| Metastases | 12 | 2 |

metastases in the hyperthermia group is not due to the use of an invasive procedure to check intra-tumour temperature in a few tumours (7), since none of the proven metastases occurred in this group.

In summary, we find the rate of proven metastasis following locally curative hyperthermia to be more than double the most generous estimate of the metastasis rate following locally curative radiation. The clinical implications for therapeutic hyperthermia are self-evident.

The basic mechanisms involved in this effect are subject to ongoing investigation, as is the possibility that combination of radiation and hyperthermia may obviate the metastatic effect of heat alone. Since, in the present study, the hyperthermia treatment group has been compared with historical controls, an extensive prospective trial is now in progress. Meanwhile, caution is required in the use of local hyperthermia alone as a form of treatment of human cancer.

The authors are grateful for the technical assistance of Mrs Barbara Clarke and Mr A. MacQuarrie. This research was supported by a Medical Research Council project grant to the Glasgow Institute of Radiotherapeutics and Oncology.

### REFERENCES

ABDELAAL, A. S. & NIAS, A. H. W. (1978) Regression, recurrence and cure in an irradiated mouse tumour. *J. R. Soc. Med.* (In Press).

CRILE, G. (1963) The effects of heat and radiation on cancers implanted in the feet of mice. *Cancer Res.*, **23**, 372.

DEWEY, W. C., THRALL, D. E. & GILLETTE, E. L. (1977) Hyperthermia and radiation—a selective thermal effect on chronically hypoxic tumour cells *in vivo*. *Int. J. Radiat. Oncol. Biol. Phys.*, **2**, 99.

DICKSON, J. A. & MUCKLE, D. S. (1972) Total body hyperthermia versus primary tumour hyperthermia in the treatment of the rabbit VX-2 carcinoma. *Cancer Res.*, **32**, 916.

DICKSON, J. A. & ELLIS, H. A. (1974) Stimulation of tumour cell dissemination by raised temperature (42°C) with transplanted Yoshida sarcoma. *Nature*, **248**, 354.

DICKSON, J. A. & ELLIS, H. A. (1976) The influence of tumour volume and degree of heating on the response of the solid Yoshida sarcoma to hyperthermia. *Cancer Res.*, **36**, 1188.

HAHN, G. M. (1974) Metabolic aspects of the role of hyperthermia in mammalian cell inactivation and their possible relevance to cancer treatment. *Cancer Res.*, **34**, 3117.

ROBINSON, J. E., WIZENBERG, M. J. & MCCREADY, W. A. (1974) Combined hyperthermia and radiation suggest an alternative to heavy particle therapy for reduced Oxygen Enhancement ratios. *Nature*, **251**, 521.

SHELDON, P. W., BEGG, A. C., FOWLER, J. F. & LANSLEY, I. F. (1974) The incidence of lung metastases in C3H mice after treatment of implanted solid tumours with X-rays or surgery. *Br. J. Cancer*, **30**, 342.

WHELDON, T. E., ABDELAAL, A. S. & NIAS, A. H. W. (1978) Growth delay analysis for cellular radiosensitivity of C3H mouse mammary carcinoma. *Br. J. Cancer* (Abst.), **38**, 178.

YERUSHALMI, A. (1976) Influence of metastatic spread of whole body or local tumour hyperthermia. *Eur. J. Cancer*, **12**, 455.